# CD19×CD3 bispecific T cell engager treatment induces remission in experimental pemphigoid disease

**DOI:** 10.1016/j.omta.2026.201825

**Published:** 2026-07-30

**Authors:** Natalie Gross, Alexander Jochimsen, Sören Dräger, Thomas Theocharis, Andreas Recke, Nina van Beek, Leon F. Schmidt-Jiménez, Steffen Krohn, Katja Klausz, Katja Bieber, Ralf J. Ludwig, Matthias Peipp

**Affiliations:** 1 Lübeck Institute of Experimental Dermatology, University of Lübeck, 23562 Lübeck, Germany; 2 Division of Antibody-Based Immunotherapy, Department of Medicine II, Christian-Albrechts University and University Hospital Schleswig-Holstein, Campus Kiel, 24105 Kiel, Germany; 3 Department of Dermatology, Allergology and Venerology, University Hospital Schleswig-Holstein, Campus Lübeck, 23562 Lübeck, Germany; 4 Institute and Comprehensive Centre for Inflammation Medicine, University of Lübeck, 23562 Lübeck, Germany

**Keywords:** bispecific T cell engagers, CD3, CD19, pemphigoid disease, bullous diseases

## Abstract

Pemphigoid diseases (PDs) are autoimmune disorders marked by autoantibodies (Aabs) against skin and mucous membrane proteins, causing mucocutaneous blistering in predominantly elderly patients. Since current therapeutics are often insufficient, PDs impose a significant morbidity and mortality burden. CD19×CD3 bispecific T cell engagers (TCEs)—originally developed for B cell malignancies—have shown promise in treatment-refractory autoimmune diseases such as systemic sclerosis and rheumatoid arthritis. To explore their potential in PDs, we tested a murine CD19×CD3 TCE in a mouse model of epidermolysis bullosa acquisita (EBA), a prototypical PD. The TCE selectively depleted B cells in the blood and bone marrow, but not the spleen, of healthy mice. Mice with clinically manifest immunization-induced EBA were randomized to receive either the CD19×CD3 TCE or control treatment upon reaching a predefined disease severity. After 13 weeks, 45% of TCE-treated mice achieved remission, vs. 23% of controls. This improvement correlated with reduced antigen-specific B cells, although total and antigen-specific IgG levels were unchanged. These findings suggest that CD19×CD3 TCEs preferentially target autoreactive B cells and may be effective at lower doses than those used in oncology. Our data support the potential of CD19×CD3 bispecific TCEs as a therapeutic strategy for PDs.

## Graphical abstract

**Figure:**
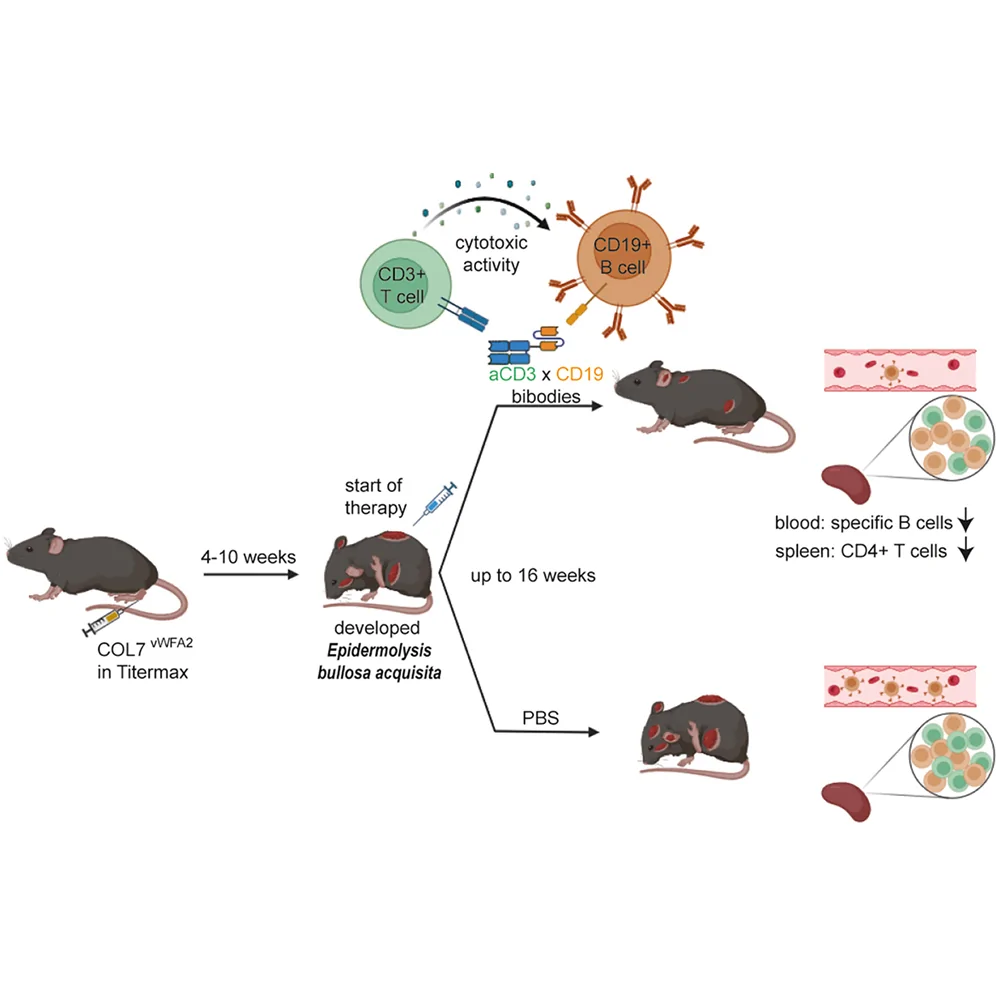

## Teaser

Gross and colleagues demonstrate that CD19×CD3 bispecific T cell engagers can selectively target autoreactive B cells and improve clinical outcomes in a mouse model of pemphigoid disease, highlighting their potential as a novel therapeutic strategy for autoimmune blistering disorders.

## Introduction

Autoimmune diseases pose a substantial medical burden. In many autoimmune diseases, pathogenic autoantibodies (Aabs) have been identified. This makes Aabs—and, consequently, Aab-producing cells such as B and plasma cells—promising therapeutic targets. Accordingly, treatments such as immunoadsorption, high doses of intravenous immunoglobulin G (IVIG), and rituximab have been established by directly removing circulating antibodies, promoting their degradation via the neonatal Fc receptor (FcRn), or depleting antibody-producing B cells.^1^^,^^2^^,^^3^ However, these therapies do not achieve consistent remission rates across all Aab-mediated diseases. Pemphigoid diseases (PDs), a group of chronic autoimmune skin blistering diseases, are one such example.^4^

In PDs, Aabs targeting antigens of the dermal-epidermal junction (DEJ) drive disease pathology by recruiting effector cells such as neutrophils, T cells, and monocytes, ultimately leading to inflammation and subepidermal blistering.^5^^,^^6^ Bullous pemphigoid, the most common subtype of PD, predominantly affects elderly individuals with an average age between 78 and 80 years, depending on the study population.^7^^,^^8^^,^^9^ Given that nonspecific immunosuppression, such as systemic corticosteroids, substantially increases morbidity and mortality in this vulnerable patient population, there remains a critical unmet medical need for safer and more effective therapies. This is underscored by the limited efficacy of the CD20-targeting antibody rituximab.^10^ Across multiple studies, rituximab has demonstrated remission rates ranging from 70% to 85%.^4^^,^^10^^,^^11^^,^^12^ Nevertheless, adverse events occurring in 20%–24% of patients and recurrence rates of approximately 20% highlight the ongoing need for more targeted approaches that effectively deplete autoreactive B cells while minimizing systemic immunosuppression.^4^^,^^13^^,^^14^^,^^15^

Two emerging strategies specifically targeting B cells are chimeric antigen receptor T (CAR-T) cells and bispecific antibodies. Both have been recently reviewed elsewhere.^16^ The efficacy of CAR-T or chimeric autoantibody receptor (CAAR-) T cells has been demonstrated in experimental models and patients, such as desmoglein-3 (DSG3)-CAAR-T cells in mucosal pemphigus vulgaris.^17^^,^^18^^,^^19^ However, high production costs,^20^ a complex manufacturing process, the need for pre-infusion conditioning with cell-depleting therapy, and—although minimal—the risk of CAR-T cell lymphomas all constrain their broad clinical application,^21^ particularly in elderly and multimorbid patients. Bispecific antibodies, such as blinatumomab, have emerged as potential therapeutics for autoimmune diseases.^22^^,^^23^^,^^24^^,^^25^^,^^26^ While conventional therapeutic IgG1 antibodies bind to a single antigen and predominantly mediate target cell killing via Fc-mediated effector functions such as complement activation, antibody-dependent cell-mediated cytotoxicity (ADCC), and antibody-dependent cellular phagocytosis (ADCP), bispecific antibodies are engineered to simultaneously recognize two distinct antigens. In the case of bispecific T cell-engaging antibodies, one domain binds a target cell-associated antigen, whereas the other recruits T cells, typically by binding to CD3. This interaction facilitates the formation of an immunological synapse, leading to T cell activation and potent target cell lysis. Numerous bispecific antibody formats have been developed, and some have received regulatory approval for clinical use, especially in hematological malignancies. Examples include blinatumomab (CD19×CD3, for relapsed/refractory B cell precursor acute lymphocytic leukemia) and teclistamab (BCMA×CD3, for relapsed/refractory multiple myeloma).^27^^,^^28^ Compared with CAR-T therapy, bispecific T cell engagers (TCEs) offer the advantage of being immediately available off the shelf. Both therapies can cause side effects such as cytokine release syndrome (CRS) and immune effector cell-associated neurotoxicity syndrome (ICANS). However, progress in biomarkers, dosing strategies, and safer constructs is improving their safety and efficacy.^29^

However, since patients with PD are often multimorbid,^9^^,^^14^^,^^15^^,^^30^^,^^31^ and therefore particularly vulnerable, thorough preclinical evaluation is essential before advancing new therapies to clinical use. To this end, we used the established immunization-induced mouse model of epidermolysis bullosa acquisita (EBA),^32^^,^^33^ which enables the investigation of therapeutic interventions in a controlled experimental setting. This preclinical model is ideal for assessing the efficacy of our CD19×CD3 TCE construct. In this model, mice are immunized with murine type VII collagen (COL7), leading to pathogenic Aab production and EBA development. The model has been extensively validated and widely used to study PD pathogenesis and evaluate potential therapeutics.^5^

To explore the concept of specifically targeting B cells in PD, we generated a murine CD19×CD3 bispecific antibody, validated its B cell-depleting activity in healthy mice, and ultimately evaluated its therapeutic efficacy in the immunization-induced EBA model.

## Results

### Design and *in vitro* characterization of CD19×CD3 TCE

**Figure 1:**
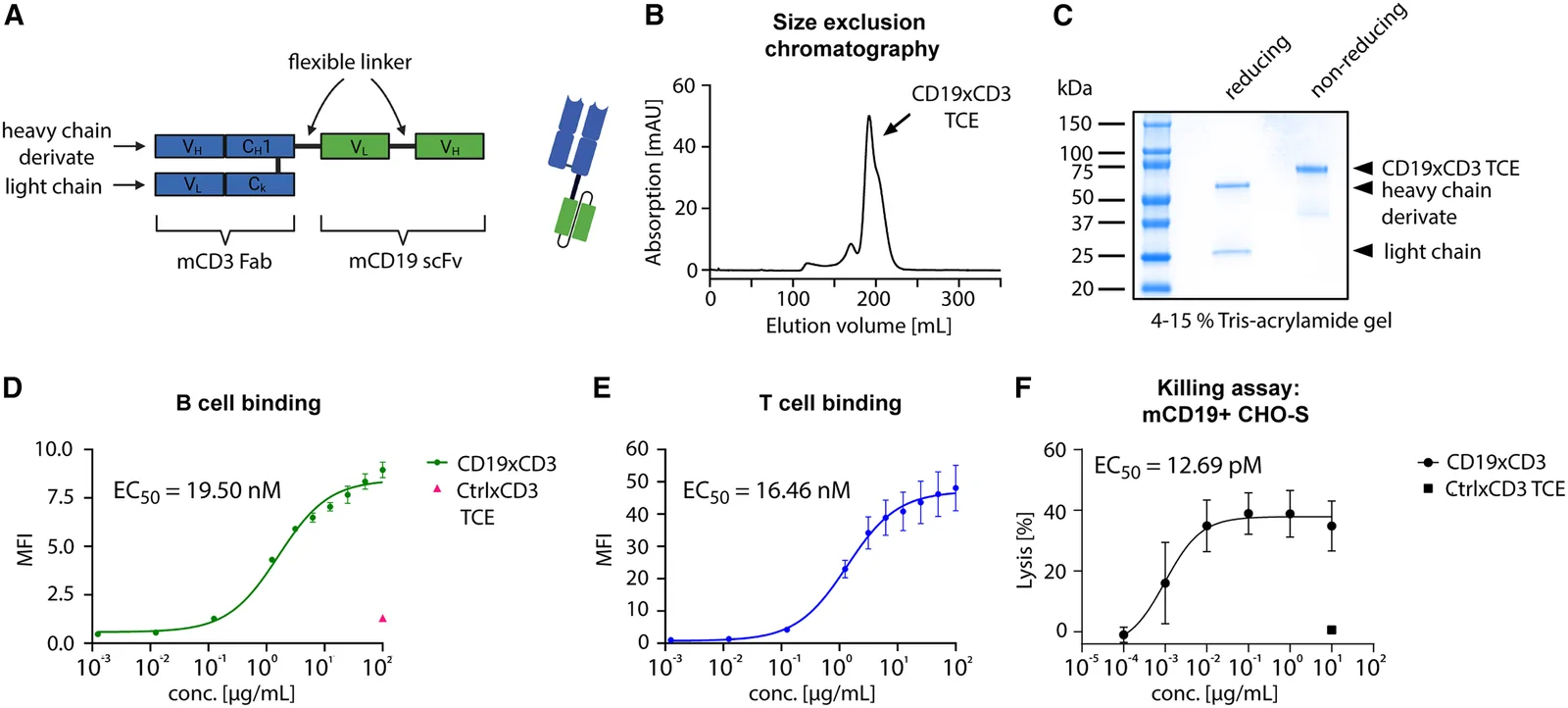
Design, production, and characterization of the CD19×CD3 TCE The CD19×CD3 TCE was produced in CHO-S cells using the MaxCyte (STX) electroporation system. The molecule was purified from cell culture supernatants by affinity chromatography (CaptureSelect CH1-XL, Thermo Fisher Scientific), followed by size-exclusion chromatography (ÄKTA Pure). (A) Schematic representation of the CD19×CD3 TCE. (B) Size-exclusion chromatography of the purified CD19×CD3 TCE. The peak at 190 mL represents the monomeric form of the molecule. (C) Coomassie blue-stained SDS-PAGE analysis under reducing and non-reducing conditions. (D) Concentration-dependent B cell binding, analyzed via flow cytometry. Murine B cells were isolated from the spleen using the Pan B Cell Isolation Kit, Mouse (Miltenyi). (E) Concentration-dependent T cell binding, analyzed via flow cytometry. Murine T cells were isolated from the spleen using the Pan T Cell Isolation Kit II, Mouse (Miltenyi). (F) CD19×CD3 TCE-mediated target cell killing, analyzed by a chromium release assay. Isolated T cells from murine spleens were used as effector cells at an effector-to-target ratio of 40:1. Transiently transfected CHO-S cells expressing murine CD19 were used as target cells. Data are presented as mean values ±SD (for binding curves) or ±SD (for killing assays) from three independent experiments. TCE, T cell engager.

To develop the bispecific CD19×CD3, a TCE format was utilized in which a Fab fragment was coupled with an scFv through a flexible linker. Here, the Fab fragment of the murine CD3 antibody (clone 145-2C11) was linked to the variable light-chain (VL) and variable heavy-chain (VH) regions of a murine CD19 antibody (clone 1D3) via glycine-serine linkers (Figure 1A). The TCE was produced in CHO-S cells via transient transfection and subsequently purified through affinity chromatography followed by size-exclusion chromatography (SEC). Re-analysis of the purified molecule using SEC revealed a major peak at the expected retention volume (Figure 1B). Furthermore, SDS-PAGE under reducing and non-reducing conditions, followed by Coomassie blue staining, confirmed purity. As anticipated, two bands corresponding to the CD3 light chain (25 kDa) and the heavy-chain derivative (55 kDa) were observed at the calculated molecular masses under reducing conditions. Under non-reducing conditions, a band for the assembled antibody was detected at a molecular mass of 75 kDa (Figure 1C). To confirm binding specificity, murine CD19^+^ B cells and CD3^+^ T cells were isolated from the spleen and incubated with varying concentrations of the antibody. The CD19×CD3 TCE exhibited comparable affinities for CD19 and CD3, with half-maximal effective concentrations (EC_50_) of 19.5 and 16.46 nM, respectively. Differences in maximum mean fluorescence intensity (MFI) values at saturation were attributed to varying antigen densities on the cell surface (Figures 1D and 1E). To evaluate the ability of the CD19×CD3 TCE to mediate T cell-dependent killing of CD19 cells, chromium release assays were performed using CHO-S cells transiently expressing murine CD19 as target cells. The TCE demonstrated efficient target cell lysis in a concentration-dependent manner, with an EC_50_ value of 12.69 pM. In contrast, the control antibody (Ctrl×CD3) showed no target cell lysis (Figure 1F). In conclusion, the CD19×CD3 TCE exhibited high-affinity binding to CD19^+^ target cells and CD3^+^ effector cells and mediated potent target cell lysis *in vitro* at picomolar concentrations.

### Doses of 1 μg CD19×CD3 TCE and above result in B cell depletion lasting 24 h

**Figure 2:**
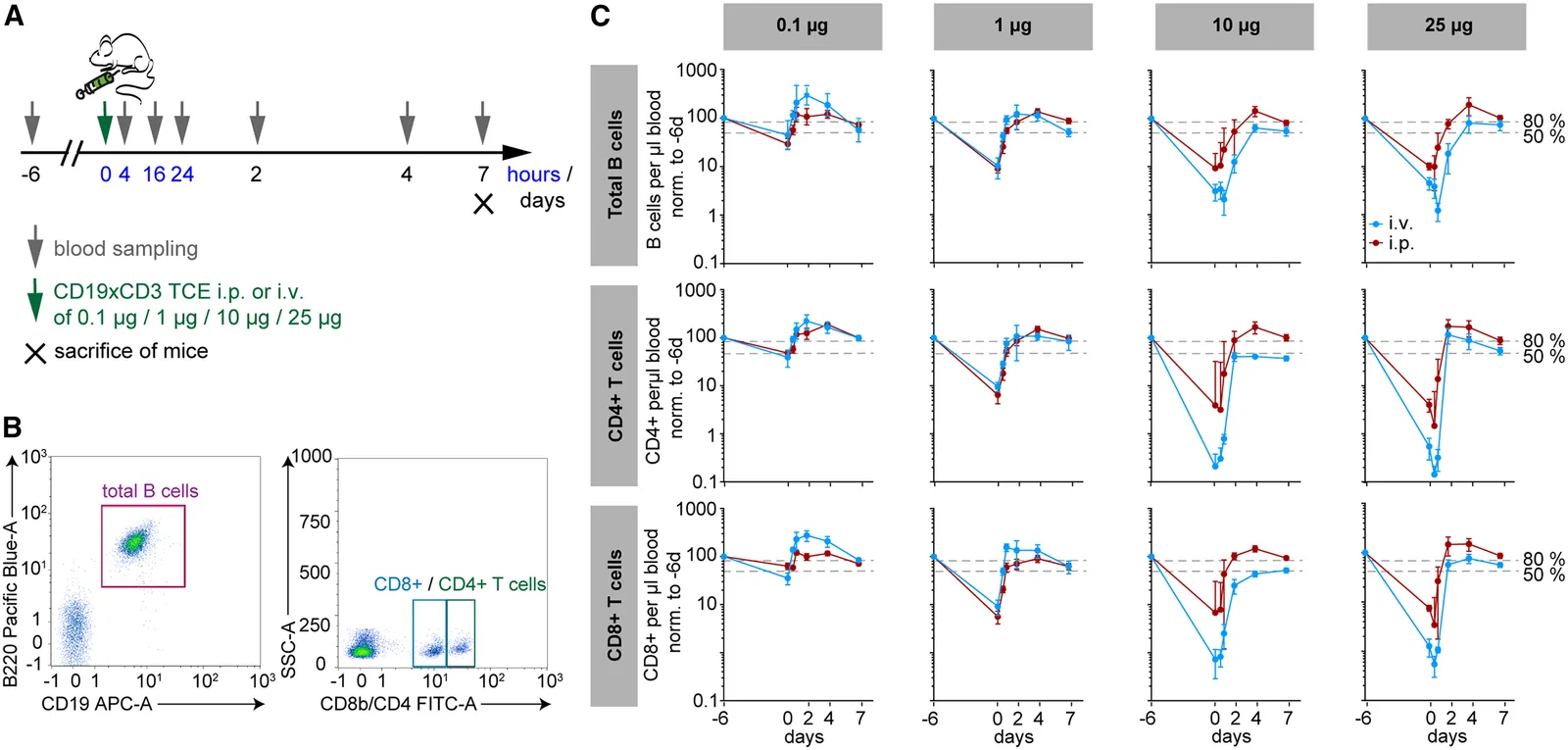
Efficient B cell depletion in peripheral blood with 1 μg of CD19×CD3 TCE Dose finding was conducted in healthy B6.S mice to find the optimal dosage and route of application of the CD19×CD3 TCE to achieve B cell depletion in peripheral blood, aiming at a relative depletion of at least 50%. (A) Schematic of the experimental setup with either intraperitoneal (i.p.) or intravenous (i.v.) injections in dosages of 0.1, 1, 10, or 20 μg. Blood was taken from the submandibular vein 6 days before and 4, 16, and 24 h and 2, 4, and 7 days after the injection of the CD19×CD3 TCE. B and T cell frequencies were measured by flow cytometry. After 1 week, the experiment was terminated, and mice were euthanized. (B) Representative flow cytometry plots for total B cells (CD19^+^B220^+^) and T cells (CD8b^+^ or CD4^+^) from one mouse at time point 0. Both cell populations were gated from single living CD45^+^ cells, and cytotoxic and helper T cells were distinguished from each other by the fluorescence intensity of flow cytometry antibodies against CD8b and CD4. (C) Time course of the number of total B cells, CD4^+^, and CD8^+^ T cells per μL blood after the injection of the CD19×CD3 TCE either i.p. (red) or i.v. (blue) in four different dosages. Values were normalized for each mouse to the baseline level. Data are represented as mean values ±SD with *n* = 3 per group. TCE, T cell engager.

Initial dose finding was performed in healthy B6.S mice, with the endpoint being the relative number of B cells and T cells to the number before injection of TCEs, with the aim of reaching at least a 50% reduction (Figures 2A and 2B). TCEs were well tolerated, as indicated by a low burden across all groups and no change in body weight (Table S1). A 50% reduction was achieved at a dose of 1 μg or higher irrespective of the application route (Figure 2C). At the lowest concentration (0.1 μg), B cell counts decreased to 29% (intraperitoneal [i.p.]) and 51% (intravenous [i.v.]). Higher doses than 10 μg only led to a slightly more pronounced depletion, with 9% (i.p.) and 13% (i.v.) for 1 μg and 15% (i.p.) and 3% (i.v.) for 10 μg. Of note, frequencies of T cells (measured by CD4^+^ and CD8^+^ cells) were also decreased in peripheral blood at doses of 1 μg and above. With doses of 10 μg and higher, T cells remained depleted for 24 h. Higher doses did not prolong the T cell depletion. However, irrespective of the dose and application route, the relative B cell count recovered after 2 days, indicating that multiple injections were needed for sustained B cell depletion. Due to the greater burden of repetitive i.v. injections and similar depleting activities of the alternative application routes, i.p. injections were selected for the subsequent experiments.

### Multiple injections of TCEs lead to sustained B cell depletion in blood and bone marrow

**Figure 3:**
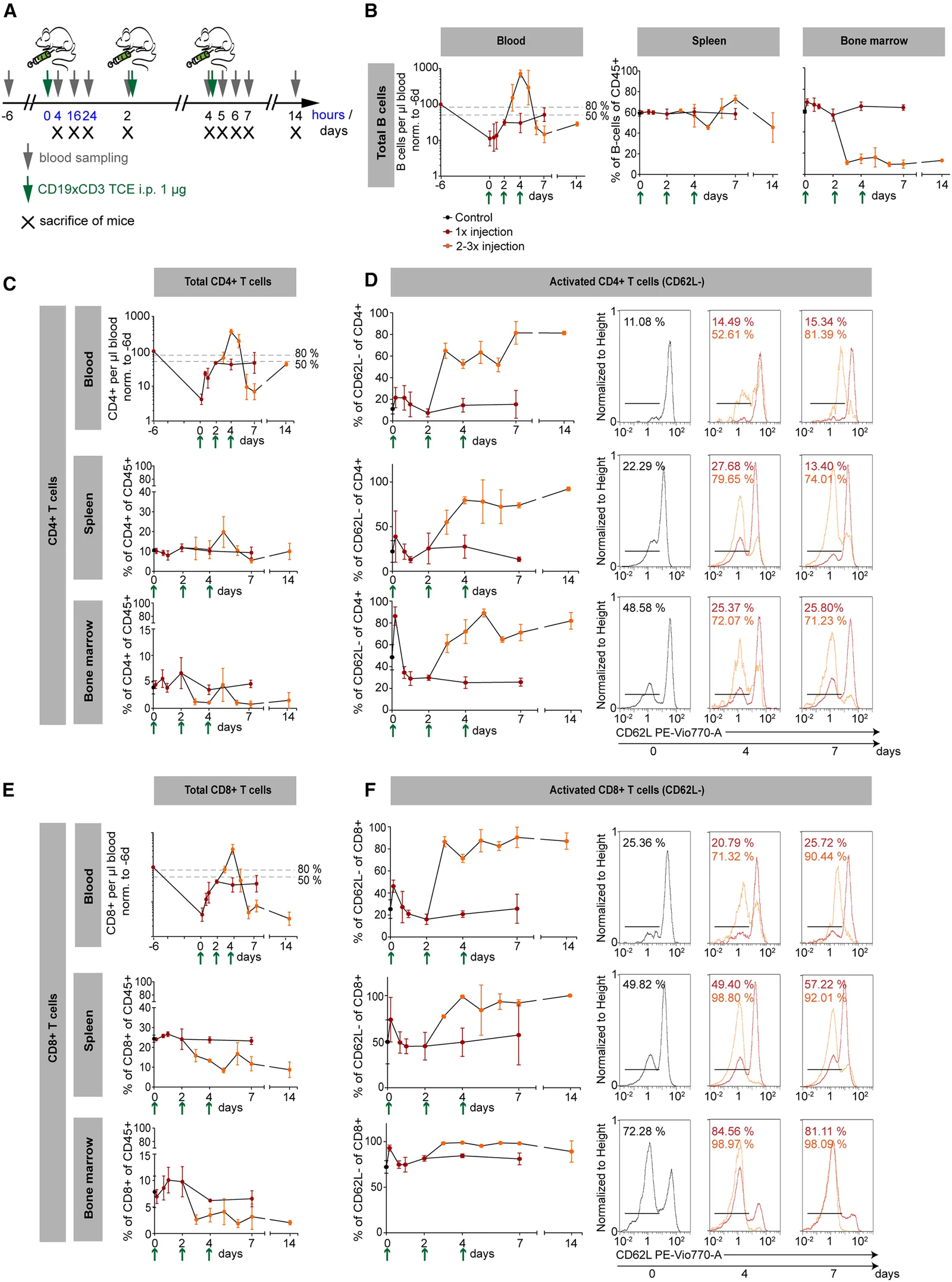
Consecutive injections of the CD19×CD3 TCE result in a stable depletion of B cells and activation of T cells in different compartments For a deeper characterization of the effect of the CD19×CD3 TCE on B and T cells in blood, spleen, and bone marrow, the TCE construct was tested in healthy B6.S mice using repeated injections of 1 μg i.p. (A) Schematic representation of the experiment: TCEs were injected on days 0, 2, and 4, and blood sampling was performed 6 days before and 4, 16, and 24 h and 2, 4, 7, and 14 days after the first injection. Mice were sacrificed at the indicated time points to analyze the B and T cells in blood, spleen, and bone marrow. (B, C, and E) Comparison of the effect of a 1× injection (red) to 2×–3× injections (orange) at the given time points (green arrows) on (B) total B cells and (C) CD4^+^ and (E) CD8^+^ T cells in blood, spleen, and bone marrow. Values in blood were normalized to the baseline level for each mouse obtained from the first blood sampling 6 days prior to the first injection. In spleen and bone marrow, B and T cells are depicted as percentages of CD45^+^ cells. (D and F) For T cells, activation was assessed by the shedding of CD62L (CD62L^−^ cells) by flow cytometry for (D) T helper and (F) cytotoxic T cells in the respective lymphatic organs and compared between the 1× and 2×–3× injection group. Respective histograms with a marked CD62L^−^ population are shown for day 0 (control group), as well as days 4 and 7, with the mean percentage of CD62L^−^ cells for each group. Activated CD62L^−^ T cells are depicted as percentages of CD4^+^ or CD8^+^ T cells. Untreated healthy B6.S mice were used to obtain control values for spleen and bone marrow, and data are represented as mean values ±SD with *n* = 3 per group. TCE, T cell engager.

To achieve prolonged B cell depletion, healthy B6.S mice received additional i.p. injections of a dose of 1 μg CD19×CD3 TCE after 2 and 4 days (Figure 3A), and, in comparison to the previous experiment, the spleen and bone marrow were additionally investigated. Weight loss and total burden were comparably low in all groups (Table S2). This time, a single injection of 1 μg of TCE led to a more pronounced B cell depletion that lasted up to 4 days in the blood. The same was obtained for CD4 and CD8 T cells. Assessment of spleen and bone marrow showed no depletion of B or T cells using a single injection. In the peripheral blood, following the second injection on day 2, a more than 7-fold increase of B cells was observed. A third injection reduced B cell counts markedly to 24% by day 7, and they remained low thereafter. In the bone marrow, B cells decreased after the second injection and remained low throughout the experiment. These findings suggest mobilization of B cells from the bone marrow into the circulation, potentially explaining the transient peripheral increase, where they were subsequently depleted by the TCE injection on day 4. (Figure 3B).

Similar to the findings obtained for B cells, the second injection led to an increase in T cells in the peripheral blood, with more than a 3-fold increase of CD4 and CD8 T cells on day 4 (Figure 3C). In the bone marrow, CD4 T cells were persistently decreased from day 7 onward. CD8 T cells in the spleen and bone marrow decreased following the second and third injections. These findings suggest that, as with B cells, T cells may transiently migrate from lymphoid tissues into the circulation following the second injection, where they are subsequently depleted by the repeated TCE administration (Figures 3C and 3E). Single injections of the CD19×CD3 TCE led to a relatively short activation of CD4 T cells in all analyzed organs, as measured by shedding of the CD62L antigen (Figure 3D). In contrast, after the second and third injections, levels of activated CD4 T cells increased to up to 8-fold higher than before TCE injection. Similar results were obtained for CD8-activated T cells in the analyzed organs (Figure 3F).

In summary, these experiments demonstrate that while B cell depletion in the peripheral blood was achieved with a single injection of 1 μg of TCE, additional depletion in the bone marrow could only be obtained by repeated injections after 2 and 4 days. Therefore, for the following experiments, 1 μg administered twice weekly was selected as the appropriate dosage to achieve sustained B cell depletion across compartments.

### Immunization-induced EBA is improved by therapeutic treatment with CD19×CD3 TCE

**Figure 4:**
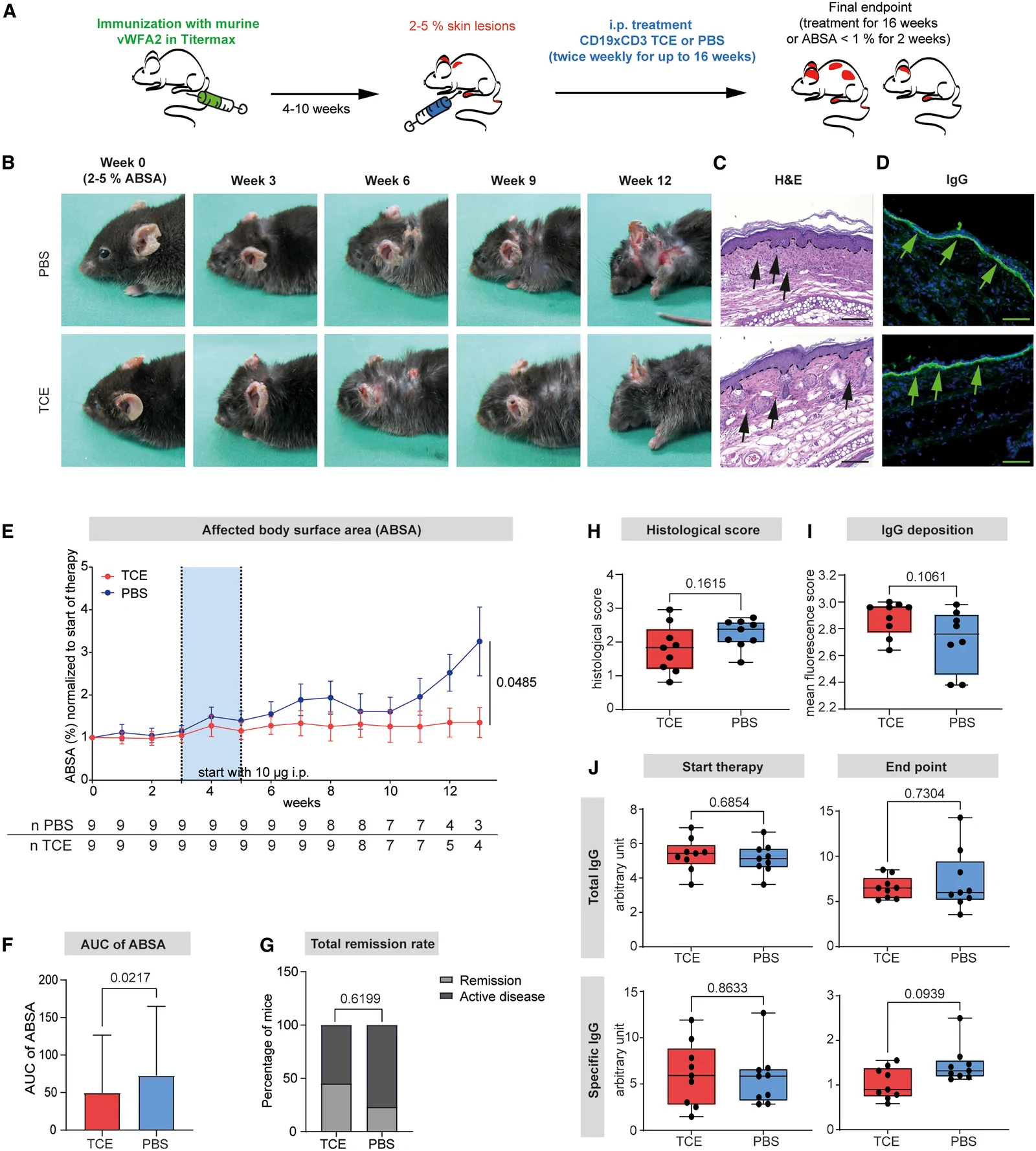
Treatment with CD19×CD3 TCE inhibits disease progression and promotes remission in an immunized mouse model of epidermolysis bullosa acquisita To evaluate the clinical efficacy of the CD19×CD3 TCE, healthy B6.S mice were immunized on day 0 by injecting 60 μg murine vWFA2 antigen dissolved in TiterMax in both hind foot pads (A). Epidermolysis bullosa acquisita (EBA) skin lesions developed after 3–5 weeks, and mice were scored weekly to assess the percentage of the affected body surface area (ABSA). If ABSA increased above 2%, mice were randomized to the treatment (CD19×CD3 TCE, i.p.) or control (PBS, i.p.) group. Treatment was performed twice weekly for up to 13 weeks or until mice achieved remission (ABSA < 1% for 2 consecutive weeks). (B) Representative pictures of both groups on the day of randomization and after 3, 6, 9, and 12 weeks of treatment. (C) Representative pictures of an H&E-stained histological section from the ear of one mouse of each group at the end of the experiment. The dotted line indicates the dermal-epidermal junction (DEJ), and the black arrows show infiltrating immune cells in the dermis. (D) Direct immunofluorescence of IgG deposition (green arrows) at the DEJ on ear sections. (E) ABSA over time, normalized for each mouse to the randomization day, is shown, with the treatment group (TCE) in red and the control group (PBS) in blue. Treatment started with 1 μg of TCE twice a week, and between weeks 3 and 5, the dosage of the TCE was increased to 10 μg per injection and continued until the end of the experiment. The number of mice per group at each time point is indicated on the bottom. (F) Comparison of the area under the curve (AUC) of the ABSA from the start of the experiment until week 13. Analysis of AUC showed that mice treated with the TCE exhibited a significant inhibition of disease progression. (G) Percentage of mice in remission of each group (TCE 45%, PBS 23%). (H) Histological score, including epidermal thickness, dermal infiltration, and split formation, and (I) IgG deposition showed no difference between the two groups. (J) Total and vWFA2-specific IgG levels in plasma at the start and individual end of therapy of each mouse showed no significant difference. Data are presented as (E and F) mean values ±SD; (H–J) medians (black line), 25^th^/75^th^ percentiles (boxes), and max/min values (error bars); or (G) stacked bars with *n* = 3–9 per group. Statistical analysis: (E) two-way ANOVA with mixed-effects model, (F) unpaired *t* test, (H–J) Mann-Whitney test, and (G) Fisher’s exact test. Scale bar: 100 μm. TCE, T cell engager.

Next, we assessed the clinical efficacy of the CD19×CD3 TCE in the immunization-induced EBA mouse model. Following EBA induction, mice that reached an affected body surface area (ABSA) of ≥2% were randomized to receive either phosphate-buffered saline (PBS) or 1 μg of TCE twice weekly. Unexpectedly, no depletion of circulating B cells was noted. Hence, after 3–5 weeks of treatment, the dosage was increased to 10 μg. Treatment was continued for up to 13 weeks or until the ABSA dropped below 1% for 2 consecutive weeks, indicating remission. At the end of the experiment, mice were euthanized, and organs were taken for further analysis (Figure 4A).

In line with previous experiments, the first signs of disease were observed 3 weeks post-immunization.^34^^,^^35^^,^^36^ In total, 18 mice met the inclusion criteria, leading to randomization of 9 mice per group. There was no difference in the change in body weight between the two groups throughout the whole experiment. The total burden increased irrespective of the treatment (Figure S2). Mice receiving PBS showed a continuous increase in the ABSA (normalized to the day of randomization), reaching a maximum mean of 3.26% after 13 weeks. In contrast, mice treated with TCEs exhibited only a marginal increase, with a peak mean ABSA of 1.35%. Overall, analysis of the area under the curve (AUC) showed a significant difference in ABSA between the two groups (Figures 4B, 4E, and 4F). Twice as many mice achieved disease remission in the TCE group (4/9; 45%) compared to the PBS group (2/9; 23%), although this difference did not reach statistical significance (Fisher’s exact test, *p* = 0.61) (Figure 4G). Early euthanasia due to disease burden was required in 3 of 9 mice (34%) receiving TCE, compared to 5 of 9 mice (56%) in the PBS group. One mouse in each group died under anesthesia, and one mouse per group completed the full 16-week treatment period (Table 1). To further characterize the safety profile of CD19×CD3 TCE treatment, we performed a comprehensive multiplex cytokine analysis of plasma samples collected from all disease model mice at the start and end of therapy. Thirteen cytokines and chemokines relevant to CRS and T cell activation—including IFN-γ, TNF-α, IL-6, IL-10, IFN-α, CCL2, CCL3, CCL4, CXCL9, CXCL10, VEGF, IL-4, and GM-CSF—were quantified and normalized to each animal’s individual baseline (Table 2). None of the measured cytokines were significantly elevated in TCE-treated mice relative to PBS controls. Canonical CRS-associated cytokines—IFN-γ, TNF-α, IL-6, and IL-10—showed no significant difference between groups (*p* = 0.6620, 0.2721, 0.5287, and 0.9273, respectively). Notably, CXCL10 was the only analyte to reach statistical significance and was significantly lower in the TCE group (*p* = 0.0426), consistent with a reduction in inflammatory disease burden rather than TCE-induced inflammation.

**Table 1:** Number of mice reaching final endpoints or undergoing early termination

	TCE	PBS
Endpoint: 2 weeks of ABSA < 1%	4/9 (45%)	2/9 (23%)
Endpoint: 16 weeks of therapy	1/9 (12%)	1/9 (12%)
Early termination: Severe burden	3/9 (34%)	5/9 (56%)
Died under anesthesia	1/9 (12%)	1/9 (12%)

Two endpoints were defined after randomization: mice either went into remission, defined as an ABSA score below 1% for 2 consecutive weeks, or completed the 16-week treatment period. Mice were subject to early termination if they exhibited a high total burden (weight loss, change in behavior and physical appearance, and ABSA score). In both groups, 9 mice were successfully randomized. The data include both the absolute number and the relative percentage of mice. TCE, T cell engager.

**Table 2:** CD19xCD3 TCE-treated mice show no side effects of a cytokine release syndrome

	TCE	PBS	*p* value
IFN-γ	3.851 ± 7.166	1.134 ± 0.4709	0.6620
IL-10	1.157 ± 1.196	1.365 ± 1.916	0.9273
CCL4 (MIP-1β)	7.166 ± 13.56	1.817 ± 1.650	0.7972
IFN-α	2.096 ± 2.610	0.7322 ± 0.8005	0.0813
CXCL9 (MIG)	1.412 ± 1.927	1.185 ± 0.9332	0.7308
CXCL10 (IP-10)	0.4364 ± 0.3701	1.156 ± 0.9498	0.0426
TNF-α	2.101 ± 2.790	6.658 ± 8.820	0.2721
IL-6	1.149 ± 0.8574	0.8265 ± 0.5682	0.5287
VEGF	5.929 ± 13.77	1.340 ± 1.154	0.8639
IL-4	40.46 ± 114.0	1.218 ± 0.5904	0.7756
CCL3 (MIP-1α)	1.187 ± 1.809	2.607 ± 2.796	0.1810
CCL2 (MCP-1)	3.656 ± 7.267	0.8570 ± 0.3999	0.4374
GM-CSF	0.7229 ± 1.127	2.135 ± 2.398	0.2685

Cytokines (IFN-γ, IL-10, CCL4, IFN-α, CXCL9, CXCL9, TNF-α, IL-6, VEGF, IL-4, CCL3, CCL2, and GM-CSF) were measured in a multiplex assay in plasma samples obtained at the start of therapy and at the end of the experiment for each mouse. Shown are the values from the end of the experiment, which were normalized to the value from the start of therapy of each individual mouse, and the respective *p* values between the treated (TCE) and non-treated (PBS) groups. Values are depicted as mean ± SD. TCE, T cell engager.

In summary, treatment with the CD19×CD3 TCE resulted in improved clinical outcomes, including higher remission rates, reduced disease progression, and decreased need for early euthanasia, supporting their therapeutic potential in PDs.

### Comparable histological findings in H&E staining and IgG deposition

At the indicated endpoint, skin samples were taken for further analysis. Sections were stained with hematoxylin and eosin (H&E), and infiltration and split formation were assessed but did not differ between groups (Figures 4C and 4H). Direct immunofluorescence staining for IgG was performed; the mean fluorescent score did not differ between the two groups (Figures 4D and 4I). Overall, histological analysis of H&E staining and IgG and C3 (Figure S3) deposition revealed similar findings in both groups.

### TCE treatment does not affect specific vWFA and total IgG levels

Total IgG and IgG against von Willebrand factor 2 (anti-mCOL7^vWFA2^ IgG) levels were measured in plasma samples throughout the treatment period. At randomization, total IgG levels were comparable between groups (5.34 U [0.95] in TCE-treated mice and 5.13 U [0.86] in PBS controls). By the individual endpoint of each mouse, total IgG levels had increased slightly in both groups, reaching 6.53 U (1.21) (TCE) and 7.22 U (3.3) (PBS). Anti-mCOL7^vWFA2^ IgG levels on day 0 were 6.06 U (3.46) in the TCE group and 5.64 U (3.58) in PBS controls. These levels declined in both groups by the study endpoint of each individual mouse to 1.04 U (0.35) and 1.46 U (0.42), respectively (Figure 4J). Taken together, CD19×CD3 TCE treatment was associated with a modest, not statistically significant reduction in pathogenic Aabs, while total IgG levels remained unaffected.

### Specific vWFA2 B cells are depleted by TCE treatment in the blood

**Figure 5:**
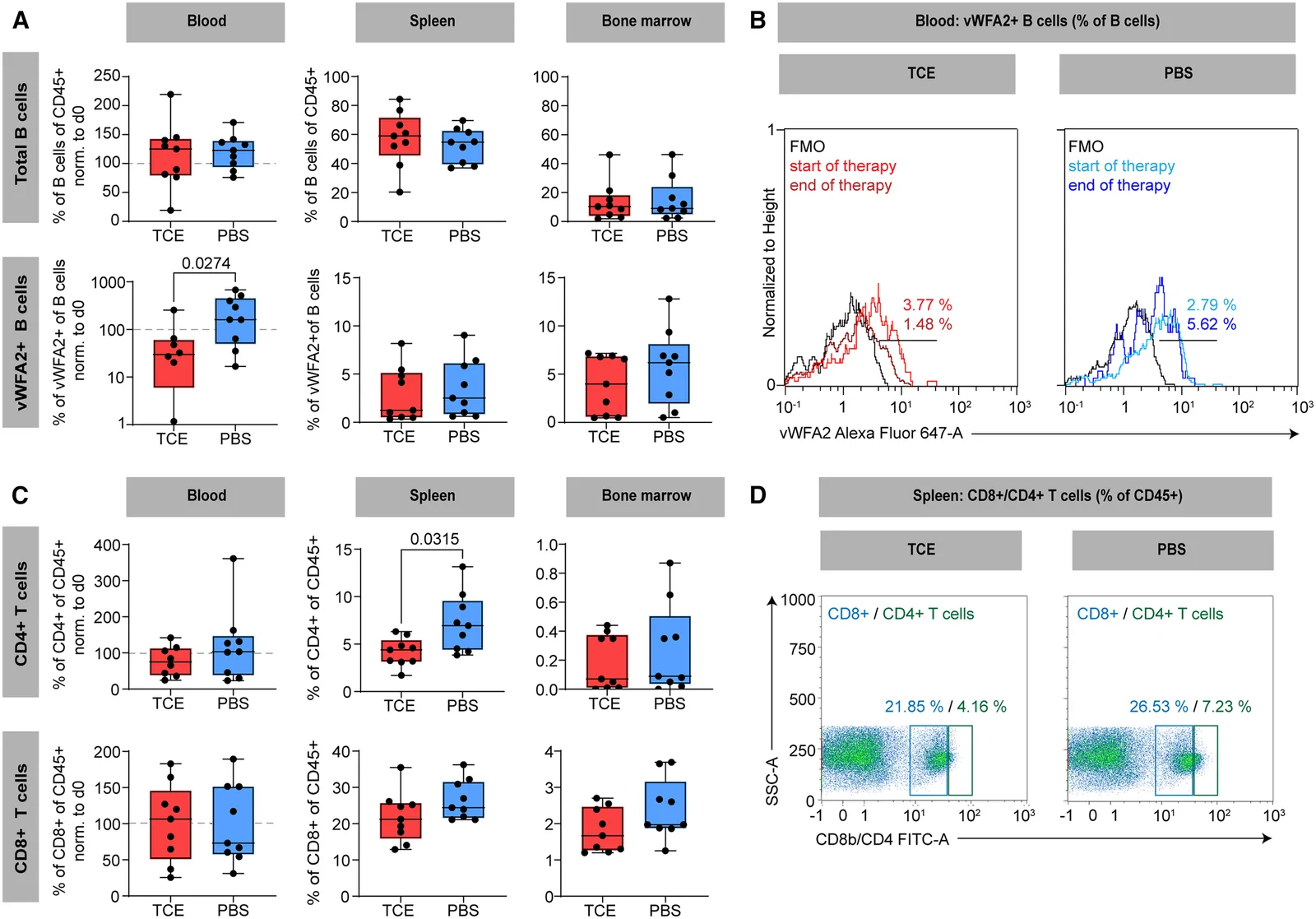
Levels of specific B cells are significantly reduced in peripheral blood after treatment with CD19×CD3 TCE Total and vWFA2-specific (vWFA2^+^) B cells, as well as T helper and cytotoxic T cells from various lymphatic organs, were analyzed by flow cytometry in mice treated with TCE in the immunization-induced model of EBA. (A) Percentages of total or vWFA2^+^ cells in blood, spleen, and bone marrow of both respective groups. Total B cells are defined as CD19^+^B220^+^ and are depicted as percentages of CD45^+^ cells, and vWFA2^+^ B cells are shown as percentages of total B cells. Values for blood are normalized to the individual values on the day of randomization. In blood, TCE treatment led to significantly fewer vWFA2^+^ B cells than the control group. (B) Flow cytometric histograms of vWFA2^+^ B cells in blood normalized to height. For each group, the plot shows the vWFA2^+^ B cell population at the start of therapy (randomization day) and at the end of therapy, as well as the corresponding FMO control. Depicted are the mean values of the vWFA2^+^ B cells. (C) Percentages of T helper (CD4^+^) or cytotoxic T (CD8^+^) cells in blood, spleen, and bone marrow of both respective groups. Both T cell populations are shown as percentages of CD45^+^ cells, and values for blood are normalized to the day of randomization. In TCE-treated mice, CD4^+^ T cells were significantly reduced in the spleen. (D) Representative flow cytometric density plots of CD8^+^ and CD4^+^ T cells gated from CD45^+^ cells of both groups. Shown are the mean percentages of T helper and cytotoxic T cells. Data are presented as medians (black line), 25^th^/75^th^ percentiles (boxes), and max/min values (error bars) with *n* = 7–9 per group. Statistical analysis: Mann-Whitney test. TCE, T cell engager.

To assess the effects of CD19×CD3 TCE treatment on B and T cell frequencies, we performed flow cytometric analysis of blood, spleen, and bone marrow. Notably, treatment reduced circulating vWFA2-specific B cells (Figures 5A and 5B). At the endpoint, relative vWFA2-specific B cell counts were 56.3% (83.9) in TCE-treated mice vs. 258.0% (231.0) in PBS controls, compared to day 0. Total and antigen-specific B cell counts in other compartments remained unchanged. Among T cells, CD4^+^ T cell frequencies were reduced in mesenteric lymph nodes (9.6% [5.2] in TCE-treated vs. 18.3% [9.5] in PBS) and spleen (4.2% [1.5] vs. 7.2% [3.1], respectively). No significant differences were observed in other compartments (Figures 5C and 5D). Overall, CD19×CD3 TCE treatment selectively depleted antigen-specific B cells in peripheral blood and reduced CD4^+^ T cell frequencies in secondary lymphoid tissues.

## Discussion

Our study demonstrates that the CD19×CD3 TCE at doses of 1 μg and above effectively depletes B cells in mice under steady-state conditions, with effects lasting several days. Repeated administration led to sustained B cell depletion in both blood and bone marrow, indicating potential for long-term, compartment-selective B cell depletion. Given the central role of autoreactive B cells in PD, we wanted to investigate whether the bispecific TCE-mediated depletion translates into therapeutic benefit in a disease model. In the immunization-induced EBA model, therapeutic TCE treatment significantly improved clinical outcomes, highlighting its efficacy in autoimmune diseases. Notably, TCE treatment also depleted circulating vWFA2-specific B cells. Taken together, these findings support the potential of CD19×CD3 TCE as a therapeutic strategy for Aab-mediated diseases, under the conditions modeled in the immunization-induced murine EBA model.

In the initial studies that led to the FDA approval of blinatumomab for Philadelphia chromosome-negative relapsed or refractory precursor B cell acute lymphoblastic leukemia (R/R B-ALL), it was continuously given as an i.v. infusion over 4 weeks of a 6 week cycle.^33^^,^^34^ A step-dose increase from 5 to 15 μg/m^2^/day was found to be best tolerated.^34^ Blinatumomab and the CD19×CD3 TCE used in this study are both designed without an Fc part, leading to significantly restricted pharmacological properties. Due to their low molecular mass (55 kDa for blinatumomab and 77.6 kDa for the CD19×CD3 TCE) and the absence of FcRn-mediated recycling, both antibodies exhibit a serum half-life of only a few hours.^37^ Based on its molecular mass of 77.6 kDa, our CD19×CD3 TCE is structurally and biophysically comparable to single-chain triple bodies, which have been reported to exhibit a serum half-life of approximately 2–4 h.^38^ This suggests that our construct may share similarly rapid systemic clearance, consistent with the observed transient B cell depletion and rapid B cell recovery. As a consequence, blinatumomab is clinically administered via continuous infusion to reduce fluctuations in systemic concentrations, thereby avoiding the risks associated with high-dose bolus injections, such as CRS or ICANS.^39^ The consequence of this pharmacokinetic limitation may in part be further supported by recent clinical data demonstrating that next-generation CD20 antibody obinutuzumab, which benefits from FcRn-mediated recycling and enhanced ADCC function, achieved deeper B cell depletion than blinatumomab in patients with lupus.^40^ However, this comparison must be interpreted with caution for two reasons. First, the specific pharmacokinetic limitations of blinatumomab’s molecular format may not be generalizable to next-generation TCE constructs engineered with improved half-life extension strategies. Second, and more fundamentally, obinutuzumab and blinatumomab deploy entirely distinct effector mechanisms: obinutuzumab mediates B cell depletion via ADCC, CDC, and direct apoptosis induction, whereas blinatumomab redirects cytotoxic T cells. The observed difference in depletion depth may therefore primarily reflect divergent effector biology rather than pharmacokinetic differences alone, and no direct mechanistic conclusions can be drawn from this comparison. Of note, hematological malignancies such as B-ALL typically involve high tumor/target cell burdens, necessitating sustained levels of antibody for high therapeutic efficacy. In contrast, autoreactive B cells in autoimmune diseases such as EBA represent only a minor fraction of the total B cell pool, which may allow for effective depletion without continuous infusion. In our initial dose-finding experiments that administered the TCE via i.p. injections three times per week, we demonstrated that this regimen was sufficient to achieve B cell depletion not only in blood but also in the bone marrow. Additionally, dosages of up to 25 μg per infusion were well tolerated and did not lead to weight loss or signs of toxicity in mice. Comprehensive multiplex cytokine profiling across 13 CRS-relevant analytes confirmed the absence of a significant cytokine release response in TCE-treated mice, suggesting a favorable safety profile at sub-oncological dosing. Nevertheless, since only endpoint measurements were performed, it could not be fully excluded that elevated cytokine levels occurred during the course of treatment that may have been resolved at later time points. Unlike in hematological cancer treatment, where continuous antibody presence is essential, the intermittent administration of the CD19×CD3 TCE appears sufficient for effective modulation of (autoreactive) B cells in autoimmune settings.

Treatment with the CD19×CD3 TCE alleviated immunization-induced EBA. Unexpectedly, total B cell counts remained largely unchanged despite clinical improvement, whereas vWFA2-specific B cells were selectively reduced.

This selective depletion may be attributable to elevated CD19 surface expression on autoreactive B cells, which could render them disproportionately susceptible to TCE-mediated killing at the doses employed.^41^ Such a mechanism would account for both the preferential elimination of autoantigen-specific B cells and the observed therapeutic efficacy in the absence of pan-B cell depletion. If confirmed, this phenomenon may represent a therapeutically meaningful selectivity, potentially reducing the immunosuppressive burden associated with global B cell ablation. The lack of sustained B cell depletion may be additionally attributed to the use of TiterMax, an adjuvant essential for disease induction in this model. TiterMax contains metabolizable oil, block copolymer CRL-8941, squalene, and a microparticulate stabilizer^36^ and induces not only the generation of mCOL7-specific B cells but also a robust local immune response. Pronounced swelling of inguinal lymph nodes and footpads (data not shown) suggests heightened B cell activation and proliferation. Such an environment may exceed the depletion capacity of TCE, unlike in healthy mice, where a single injection is sufficient to markedly reduce B cell numbers. These findings suggest that in activated immune environments, such as those induced by TiterMax administration or during active disease flares, the depletion capacity of bispecific antibodies can be limited. This highlights the challenge of B cell-directed therapies in the context of ongoing immune activation and underscores the importance of evaluating treatment efficacy in disease-relevant models. Consistent with this, pre-treatment with a CD20-targeting antibody 1 day prior to immunization attenuated antigen-specific CD4^+^ T cell responses in SJL/J mice, highlighting the broader immunomodulatory consequences of timely targeting of autoreactive B cells.^33^

The limited pharmacological properties of Fc-lacking bispecific antibodies, especially significantly reduced serum half-life, necessitate continuous administration to maintain therapeutic levels. To address this, most bispecific antibodies in clinical development are engineered as IgG-like molecules, such as the BCMA×CD3 antibodies linvoseltamab and teclistamab, which benefit from prolonged bioavailability by FcRn-mediated recycling.^42^^,^^43^ However, beyond systemic persistence, other factors such as the ability of an antibody to penetrate tissues and reach target cells in their specific niches might be equally if not more critical, particularly in autoimmune diseases, where autoreactive B cells may reside in peripheral tissues such as lymphoid organs. In this context, smaller molecules, such as the CD19×CD3 TCE, may offer a distinct advantage in accessing otherwise poorly perfused tissues or compartments.^44^ Consequently, future therapeutic strategies should not only focus on clinical efficacy but also on understanding the biological underpinnings of each disease, including the localization and phenotype of pathogenic B cell populations. Comparative studies of both antibody formats (IgG-like vs. non-IgG-like) and B cell-targeting specificities will be crucial. Importantly, multiple bispecific TCEs that have received FDA approval for hematological malignancies are currently clinically repurposed for the treatment of autoimmune entities, such as teclistamab, highlighting the expanding therapeutic potential of this class of immunotherapies.^23^

In addition to antibody-based strategies such as TCE, other immunotherapeutic approaches, including CD19-directed CAR-T cells, have recently demonstrated high efficacy in autoimmune diseases such as systemic lupus erythematosus (SLE).^40^ While CAR-T cell therapies enable deep and long-lasting B cell depletion, resulting in an effective reset of the B cell compartment, their clinical application is more complex due to individualized manufacturing and potential safety concerns.^45^ In contrast, TCEs represent an off-the-shelf approach with adjustable dosing, potentially resulting in improved safety profiles. Therefore, TCEs may serve as a complementary strategy, particularly in clinical settings where CAR-T cell therapy is not feasible.

Despite the observed clinical improvement in EBA mice, mCOL7^vWFA2^ Aab titers were unexpectedly not significantly reduced following CD19×CD3 TCE treatment. This finding is in line with the known resistance of long-lived plasma cells to CD19-targeted therapies. As terminally differentiated B cells, plasma cells typically lack surface expression of CD19 and are thus not targeted or eliminated by CD19×CD3 bispecific TCEs.^46^^,^^47^^,^^48^ In addition, previous studies have shown that plasma cells induced by vaccination can persist for over a decade, and CD20-directed therapies do not affect pre-existing humoral immunity, so that antibody titers against tetanus, measles, mumps, and rubella remain unchanged.^49^ Although CD19 is expressed on a subset of plasmablasts and early plasma cells,^50^ targeting CD19 alone may not suffice to eliminate the full pool of Aab-producing cells. These findings suggest that combining CD19-directed therapies with other molecules targeting plasma cell antigens, such as BCMA, could further enhance therapeutic efficacy in Aab-driven diseases. Recent clinical case reports also support this combinatorial approach,^51^ a strategy that can be tested in subsequent studies.

This study has several limitations. The herein presented strategy relies on rolling enrollment, with treatment initiated individually once each mouse reaches the 2% ABSA threshold. When interim monitoring indicated insufficient efficacy of the initial TCE dose, the dose was escalated immediately for all enrolled mice. In retrospect, a predefined dose-escalation time point relative to each mouse’s individual treatment start would have been methodologically preferable. Additionally, the small group size of nine mice per group limits statistical power, as reflected by the non-significant difference in remission rates despite a numerically meaningful difference. Future studies with larger cohorts and predefined dosing strategies are needed to confirm these findings and to directly benchmark CD19×CD3 TCE treatment against established B cell-depleting approaches. Accordingly, the present study should primarily be interpreted as a preclinical proof-of-concept investigation rather than a definitive comparative efficacy study.

In summary, we demonstrate that selective B cell depletion using the CD19×CD3 TCE is a promising strategy for treating Aab-mediated autoimmune diseases such as PD. Moreover, the therapy was well tolerated and could potentially be applied even in vulnerable patients. Our study may serve as a blueprint for related conditions, as the antigen specificity of TCEs can, in principle, be adapted to match the unique immunopathology of PD and other autoimmune diseases.

## Materials and methods

### Design, expression, and purification of mCD19×CD3 TCE

To generate the bispecific CD19×CD3 TCE in a bibody format, two plasmids for secretion (pSEC) encoding the heavy-chain derivative and the light chain were designed. For the heavy-chain derivative, the VH of the anti-mCD3 antibody clone 145-2C11^52^ and the constant heavy 1 (CH1) domain were fused to an anti-mCD19 scFv coding sequence (clone 1D3) via two flexible glycine-serine linkers. Plasmid DNA was amplified and purified using the Nucleo Bond 2000 EF (Macherey-Nagel, Dürren, Deutschland), and the correct sequences were confirmed by Sanger sequencing (Institute of Clinical Molecular Biology, Kiel, Germany). The TCE was produced in CHO-S cells using the MaxCyte STX large-scale electroporation system, following the manufacturer’s recommendations. For purification from cell culture supernatant, the Capture Select IgG-CH1-XL affinity matrix (Thermo Fisher Scientific) was used, followed by SEC performed with the ÄKTA pure system (Cytiva). As an isotype control, an antibody targeting murine CD3 (clone 145-2C11) and an irrelevant antigen (Ctrl×CD3) were generated in the same molecular format.

### SDS-PAGE analysis

2 μg of purified protein were loaded on a precast 4%–15% Tris-acrylamide gel (Mini-PROTEAN TXN, BioRad) under reducing and non-reducing conditions and stained overnight with Coomassie brilliant blue staining solution (Carl Roth, Karlsruhe, Germany).

### Transient transfection of CHO-S cells

For *in vitro* cytotoxicity assays, murine CD19-expressing CHO-S cells were generated via transient transfection. Therefore, the MaxCyte STX large-scale electroporation system was used following the manufacturer’s recommendations. 24 h post-electroporation, surface expression of murine CD19 was verified by flow cytometry.

### Assessment of *in vitro* cytotoxicity

Cytotoxicity assays were performed as described.^53^ In brief, target cells were labeled with 50 μCi of ^51^Cr for 2 h followed by three washes with culture medium. For effector cell preparation, murine T cells were isolated from the spleen using the Pan T Cell Isolation kit (Mouse) (Miltenyi Biotec) following the manufacturer’s recommendations. Effector cells, culture medium, and antibody were added to a round-bottom microtiter plate. The assays were initiated by dispensing the labeled target cells, i.e., murine CD19-expressing CHO-S cells, at an effector-to-target (E:T) cell ratio of 40:1 in a total volume of 200 μL. After incubation for 18 h at 37°C, ^51^Cr release from triplicate samples was measured in counts per minute (cpm) using the MicroBeta TriLux 1450 liquid scintillation and luminescence counter (PerkinElmer/Revvity). The percentage of cellular cytotoxicity was calculated using the following formula: % specific lysis = (experimental cpm – basal cpm)/(maximal cpm – basal cpm) × 100. Maximal cpm was determined by adding Triton X-100 to target cells, and basal release was determined in the absence of antibody or effector cells.

### Animal experiments

Immunization-EBA-susceptible B6.SJL-H2s C3c/1CyJ (B6.S) mice^33^ were originally obtained from the Jackson Laboratories (Stock 000966; Bar Harbor, ME, USA) and bred at the animal facility of the University of Lübeck, Germany. For the experiments, sex-matched adult mice (>8 weeks old) were used. The mice were given standardized mouse chow and acidified drinking water provided *ad libitum* and maintained on a 12-h light/dark cycle. All clinical examinations were performed under anesthesia, using i.p. administration of a ketamine (100 μg/g body weight, Sigma-Aldrich, Germany) and xylazine (15 μg/g body weight, Sigma-Aldrich, Germany) mixture unless otherwise specified. Blood sampling was performed on awake animals by puncture of the vena facialis following the recommendations of the GV-SOLAS (Freiburg, Germany). Mice were euthanized under deep anesthesia, followed by complete exsanguination and cervical dislocation. All animal experiments were conducted as per the European Community rules for animal care and were approved by the governmental administration (V242-49267/2019 (80–7/19), Ministry for Energy, Agriculture, the Environmental and Rural Areas) and conducted by certified personnel.

### Dose-finding studies of TCE in healthy mice

Dosing was performed in healthy B6.S mice with *n* = 3 per group. After initial blood sampling 6 days prior, CD19×CD3 TCEs were dissolved in PBS and injected in four different concentrations (0.1, 1, 10, and 25 μg per mouse) either i.p. or i.v. Weight was recorded at days −6 and 0 and at the end of the experiment, with day 0 being the day of TCE administration. Blood was also taken on day −6 and 4, 16, and 24 h and 2, 4, and 7 days after antibody injection. After 7 days, mice were euthanized. In a second set of experiments, blood sampling was again performed at the previously indicated time points, this time extended to 14 days. Application of 1 μg of TCE was repeated after 48 and 96 h. In this experiment, mice were euthanized at different time points to investigate B cell depletion across different immunological compartments.

### Immunization-induced EBA and preclinical study protocol

For induction of experimental EBA, B6.S mice were immunized with 120 μg of the vWFA2 domain of COL7 (mCOL7^vWFA2^) mixed in a 1:1 ratio with TiterMax by injecting 60 μL of the vWFA2/TiterMax mixture into each footpad of the hind legs, as previously published.^54^ Clinical evaluations and total burden score (considering weight loss and changes in behavior and physical appearance), and clinical EBA severity, expressed as ABSA, were carried out weekly by a person unaware of the treatment.^55^^,^^56^ Within 10 weeks following immunization, when mice reached an ABSA score of ≥2%, they were randomly assigned to either the treatment group (receiving TCE; *n* = 9) or the control group (receiving PBS; *n* = 9). The TCE group was treated with i.p. injections of 1 μg per injection, and within 3–5 weeks, the dose was increased to 10 μg. Treatment was performed twice weekly for up to 16 weeks or until mice reached remission, classified as an ABSA <1% for 2 consecutive weeks. Blood samples were taken 1 week prior to immunization, at randomization, and every 2 weeks thereafter. After 13 weeks, all remaining mice (*n* = 5) were euthanized, and blood, skin, and organs were taken for further evaluation. For assessment, relative clinical scores were calculated by comparing each week’s score to the initial score at the start of treatment (weekly score/initial score), the overall disease severity was quantified as the cumulative relative clinical score over time (AUC), and remission rates were determined.

### Histology

For histology, skin and ear samples were fixed in 3.7% paraformaldehyde and embedded in paraffin. H&E staining was performed on 4- to 5-μm-thick sections following standard protocols. The histological score was assessed by combining the individual epidermal thickness and the split formation and infiltration of immune cells by an investigator unaware of the applied treatments.^57^ To detect tissue-bound rabbit IgG and murine C3, direct immunofluorescence microscopy was performed as described.^6^ Briefly, frozen sections were prepared from 6-μm-thick skin and ear sections and incubated with goat anti-rabbit antibodies reactive with rabbit IgG (Dako Deutschland, Hamburg, Germany) and murine C3 (MP Biomedicals, Keyseberg, France). Sections were labeled with fluorescein isothiocyanate (FITC), and the respective fluorescence intensity was evaluated by a blinded investigator using a semi-quantitative assessment method.

### Flow cytometry

Flow cytometry analysis was performed in independent experiments using either a Navios flow cytometer (Beckman Coulter) with data analyzed in Kaluza Analysis software (Beckman Coulter) or a MACSQuant Analyzer 10 (Miltenyi Biotec; Bergisch Gladbach, Germany) with data analyzed in MACSQuantify Software (v.2.13; Miltenyi Biotec). Each experiment was conducted entirely on one of the two platforms. For concentration-dependent binding analysis of the CD19×CD3 TCE, 2 × 10^5^ MACS isolated murine B or T cells (C57BL/6) were washed in PBS containing 1% bovine serum albumin (BSA) and 0.1% sodium azide (PBA buffer). Next, cells were incubated with the protein at varying concentrations on ice for 60 min and washed three times with 200 μL PBA buffer. Finally, cells were stained with a secondary anti-human-kappa-FITC antibody (SouthernBiotech) on ice for 30 min, washed three times with 200 μL PBA buffer, and resuspended in 300 μL PBA buffer. For flow cytometric analyses of cell composition of blood, spleen, and bone marrow, single-cell suspensions were prepared: in short, spleens were gently ground between two slides in DPBS (Life Technologies, Carlsbad, CA, USA), and cells were filtered through a 70 μm cell strainer. Bone marrow was isolated from the femur and tibia, and cells were flushed out using a 27G syringe and PBA buffer.^58^ Erythrocytes were lysed, and live/dead staining was performed using the fixable viability dye BD620 or 780 (BD Bioscience, Becton, New Jersey, USA). After a Fc-blocking step (Miltenyi Biotec) of 10 min on ice, cells were stained for 20 min with the following antibodies: Pacific-Blue, VioGreen, FITC, PE-Vio770, and APC-conjugated anti-mouse B220 (clone: RA3-6B2); CD45 (30F11); CD4 (GK1.5); CD8 (H35–17.2); CD62L (MEL14-H2.100); and CD19 (REA749), from Biolegend (San Diego, CA, USA) or Miltenyi Biotec. Cells were gated for leukocytes (FSC-A and SSC-A) and singlets (FSC-H compared with FSC-A). Single cells were then distinguished as alive or dead. Only living cells were further gated on their positive CD45 expression and then the positive B cell (B220 and CD19) and T cell (CD4 and CD8) populations, as well as activated T cells (CD62L negative) (Figure S1). For specific vWFA2^+^ B cells, recombinant mCOL7^vWFA2^ was labeled with an Antibody Labeling Kit Alexa Fluor 647 (Invitrogen, Waltham, MA, USA) following the manufacturer’s instructions. For flow cytometric staining, labeled mCOL7^vWFA2^-Alexa Fluor 647 was used in a pre-tested concentration alongside the above-listed antibodies, and specific B cells were gated from the positive B cell population. Positive vWFA2^+^ B cells were determined by comparing the fluorescence signal to an FMO control from the corresponding mouse.

### ELISA

Plasma levels of circulating total and autoreactive anti-mCOL7^vWFA2^ IgG of immunized mice were determined by ELISA using mouse quantification sets (Bethyl, Montgomery, TX, USA) following the manufacturer’s protocol with minor modifications for the autoreactive antibodies.^59^^,^^60^ In detail, each well was coated with 250 ng recombinant mCOL7^vWFA2^ in coating buffer. After blocking, diluted samples were added and incubated for 60 min. Bound antibodies were detected by HRP-conjugated goat anti-mouse antibodies (Bethyl) and tetramethylbenzidine (Invitrogen). The enzymatic color reaction was stopped by adding 2 M sulfuric acid (Carl Roth), and the change in OD was measured with a GloMax Discover Microplate Reader photometer (Promega, Walldorf, Germany) at 450 nm. Standard reference curves were established by using the provided mouse reference sera (Bethyl).

### Blood cytokine analysis

To test for a possible side effect of a CRS in the TCE-treated group in the immunization-induced EBA mouse model, 13 cytokines were measured by a multiplex assay in plasma obtained at the start of therapy and at the individual end of the experiment from each mouse. The assay was performed using the LEGENDplex Mouse Cytokine Release Syndrome Panel (BioLegend) including the cytokines IFN-γ, IL-10, CCL4, IFN-α, CXCL9, CXCL9, TNF-α, IL-6, VEGF, IL-4, CCL3, CCL2, and GM-CSF according to the manufacturer’s instructions.

### Statistical analysis

For graphical and statistical analyses, GraphPad Prism 10 software (GraphPad Software, San Diego, CA, USA) was used. Applied tests and confidence intervals are indicated in the respective text and figure legend. *p* < 0.05 was considered statistically significant. Sample size calculation for animal experiments was performed using SigmaPlot 13.0 (Systat Software) considering the percentage of EBA-affected body area at the end of the experiment as the primary endpoint, along with the remission rates. The calculations assumed an expected standard deviation of 45% in the immunization-induced EBA model, an alpha level of 5%, a power of 85%, and a minimum detectable difference from the positive control of 50%.

## Data and code availability

The data supporting the findings of this study are available from the corresponding author upon reasonable request.

## Acknowledgments

This research was funded by the Cluster of Excellence “Precision Medicine in Chronic Inflammation” (EXC 2167), the Research Training Group “Defining and Targeting Autoimmune Pre-Disease” (GRK 2633), and the collaborative research center “Pathomechanisms of Antibody-mediated Autoimmunity” (CRC 1526), all from the Deutsche Forschungsgemeinschaft and the Schleswig-Holstein Excellence-Chair Program from the State of Schleswig-Holstein. We thank the Institute of Clinical Molecular Biology in Kiel for providing Sanger sequencing as supported in part by the DFG Clusters of Excellence “Precision Medicine in Chronic Inflammation” and “ROOTS.” We thank T. Naujoks, Dr. D. Langfeldt, and Dr. B. Löscher for technical support. The graphical abstract was created using BioRender.com.

## Author contributions

N.G., A.J., and S.D. contributed to the investigation, formal analysis, and writing of the initial draft. N.G. was responsible for the visualization. T.T. was involved in the investigation and contributed to critical revision of the manuscript. A.R. provided resources and participated in the critical revision, while N.v.B., L.F.S.-J., and S.K. also contributed to the critical revision of the writing. K.K. was responsible for methodology, supervision, and critical revision of the manuscript. K.B. contributed to conceptualization, investigation, methodology, supervision, and critical revision. M.P. and R.J.L. were involved in conceptualization, resource provision, methodology, funding acquisition, supervision, and critical revision of the manuscript.

## Declaration of interests

Nina van Beek has a joint project and a patent with Euroimmun and has received speaker honoraria from Fresenius Medical Care.

## Declaration of generative AI and AI-assisted technologies in the writing process

During the preparation of this work, the authors used ChatGPT 4o (OpenAI, San Francisco, CA, USA) in order to improve language and readability. After using this tool, the authors reviewed and edited the content as needed and take full responsibility for the content of the publication.

## Supplemental information

Document S1. Figures S1–S3 and Tables S1 and S2

Document S2. Article plus supplemental information
